# Cell Scaffolds for Bone Tissue Engineering

**DOI:** 10.3390/bioengineering7040119

**Published:** 2020-09-30

**Authors:** Kazutoshi Iijima, Hidenori Otsuka

**Affiliations:** 1 Faculty of Engineering, Yokohama National University, 79-5 Tokiwadai, Hodogaya-ku, Yokohama 240-8501, Japan; iijima-kazutoshi-mh@ynu.ac.jp; 2 Department of Applied Chemistry, Faculty of Science, Tokyo University of Science, 1-3 Kagurazaka, Shinjuku-ku, Tokyo 162-8601, Japan; 3 Department of Chemistry, Graduate School of Science, Tokyo University of Science, 1-3 Kagurazaka, Shinjuku-ku, Tokyo 162-8601, Japan

**Keywords:** tissue engineering, cell scaffold, mesenchymal stem cells, electrospun nanofiber, silica nonwoven fabrics

## Abstract

Currently, well-known surgical procedures for bone defects are classified into four types: (1) autogenous bone graft transplantation, (2) allogeneic bone graft transplantation, (3) xenogeneic bone graft transplantation, and (4) artificial bone graft transplantation. However, they are often risky procedures and related to postoperative complications. As an alternative, tissue engineering to regenerate new bone often involves the use of mesenchymal stem cells (MSCs), derived from bone marrow, adipose tissues, and so on, which are cultured into three-dimensional (3D) scaffolds to regenerate bone tissue by osteoinductive signaling. In this manuscript, we provide an overview of recent treatment of bone defects and the studies on the creation of cell scaffolds for bone regeneration. Bone regeneration from bone marrow-derived mesenchymal stem cells using silica nonwoven fabric by the authors’ group were provided. Potential application and future direction of the present systems were also described.

## 1. Introduction

To date, clinical operations, including knee osteoarthritis, bone grafting, and fracture repair are performed throughout the worldwide. A substantial percentage is occupied by the elderly, whose numbers are expected to double in the next 25 years [1]. Fractures caused by osteoporosis smite roughly one in three women and one in five men over the age of 50, and are principal reasons for suffering in elderly populations [2]. Other percentages are caused by accident- or sports-related injuries and treatment for bone tumors. If the defect site is small, it will recover spontaneously. When the defect size is greater than the healing capacity of osteogenic tissues, treatment with bone substitute or bone graft will be needed. Recently, bone regeneration using mesenchymal stem cells has received much attention due to its high therapeutic effect and safety. In this manuscript, we provide an overview of recent treatment of bone defects and the studies on the creation of cell scaffolds for bone regeneration. Bone regeneration from bone marrow-derived mesenchymal stem cells using silica nonwoven fabric by author groups were provided. Potential application and future direction of the present systems were also described.

## 2. Structure of Natural Bone

**Figure 1:**
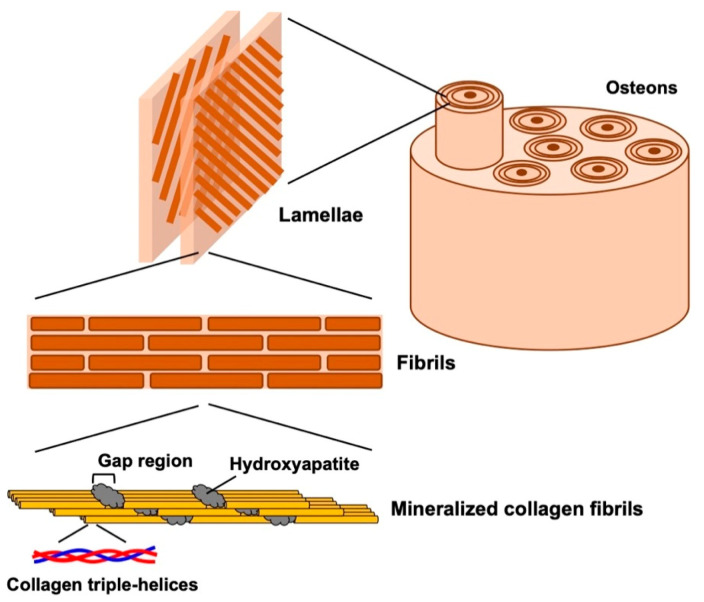
Hierarchical structural organization of bone.

Bones play important functional roles, such as providing mechanical support for locomotion, protecting organs, and controlling mineral homeostasis. Furthermore, hematopoiesis mainly occurs within bone marrow. Hierarchical structural organization of bones are shown in Figure 1. Bone tissue mainly consists of organic component, type I collagen, and inorganic component, hydroxyapatite (HAp), and show excellent toughness and stiffness by complexing at the nanoscale [3,4]. These excellent natural organic–inorganic composites at the nanoscale are formed by mineralization of HAp nanocrystals in a gap region containing acidic amino acids between bundles of type I collagen [5,6,7]. Collagen fibers with HAp are assembled into lamellae and form osteon with blood veins.

## 3. Treatment of Bone Defects

In the treatment of bone defects caused by various bone diseases, such as trauma, bone tumor, and chronic joint disease, reconstruction/compensation of bone tissue is required. Current treatments are mainly classified into three types: (1) autogenous bone graft transplantation [8], (2) allogeneic bone graft transplantation [9], (3) xenogeneic bone graft transplantation [10], and (4) artificial bone graft transplantation [11]. Autologous bone grafting is a procedure in which bone fragments collected from the patient’s own iliac or fibula and are transplanted to the bone defect lesion. Since autologous bone graft is derived from living bones, the graft containing osteogenic cells, such as osteoblasts has both osteoinductivity and sufficient mechanical properties. However, adverse events related to the autograft harvest cannot be ignored [12]. Allogeneic bone transplantation is to transplant a bone fragment from a donor into a bone defect lesion. Since it uses the bone tissues of others, mostly from cadaver—ethical issues, supply stability, and risk of infection remain [9]. Xenogeneic bone graft transplantation is to transplant a processed bone that is taken from another species, such as bovine [10]. Although deproteinization is an indispensable process to prevent immune dejection and infection, the processed xenograft exhibits limited osteoinduction activity [13]. In artificial bone graft transplantation, various materials, such as aluminum oxide (alumina) [14], calcium phosphate, including hydroxyapatite (HAp) [15], beta-tricalcium phosphate (β-TCP) [16], carbonate apatite [17], and bioactive glass [18,19,20], have been developed and examined. Among them, β-TCP showed excellent biocompatibility and absorbability. Bioactive glass is a silica-based material containing additives, such as sodium oxide, calcium, and phosphate. It has been accepted as an implantable scaffold for bone regeneration due to its osteoconductive, osteoproductive, and osteoinductive properties. Bioactive glass is used, not only as a single substance, but also as a composite with other materials. For example, the osteoconductivity of polyethersulfone nanofibers was reinforced by mixing with bioactive glass nanoparticles [21]. However, these artificial bones are not enough to fill a large defect. Furthermore, inorganic/organic composites as scaffold is discussed in Section 5.

## 4. Bone Tissue Regeneration Using Mesenchymal Stem Cells (MSCs)

Bone tissue regeneration using mesenchymal stem cells (MSCs) has been attracting much attention [22]. MSCs are isolated from bone marrow [23], adipose tissue [24], umbilical cord [25], and dental pulp [26], much less invasively than autologous bone tissue. Further, it can be relatively easily cultivated and propagated in vitro before transplantation. In Table 1, the advantages and disadvantages associated with cell sources [27]. Bone marrow-derived mesenchymal (BM)-MSC is mainly and intensively used for bone tissue regeneration from MSC, and more studies are needed to test use of MSCs from other sources in bone repair. MSCs can contribute regeneration of bone defect through direct and indirect effects [22]. MSCs can differentiate into mesenchymal tissues including bone, cartilage, tendon, muscle, and so on [28]. As a direct effect, MSCs develop to become bone forming cells, osteoblasts secreting and mineralizing the bone matrix. MSCs from any of the above sources have been shown to be capable of differentiating into osteoblasts [22,23,24,25,26]. MSCs may also contribute to bone regeneration through indirect effects, by producing cytokines, growth factors, and regulating vascularization and modulating inflammation [22]. It has been demonstrated that vascular endothelial growth factor (VEGF) secreted from MSCs promoted bone regeneration with angiogenesis [29]. There are two methods for bone regeneration using MSCs: a method of constructing bone tissue from MSCs in vitro and transplanting it, and a method of transplanting MSCs to induce bone regeneration in vivo. In the former method, MSCs are cultured in a bone differentiation-inducing medium containing dexamethasone, ascorbic acid, and β-glycerophosphate for a week to differentiate into osteoblasts, and formed bone tissue is transplanted [30]. In the latter method, MSCs are transplanted and differentiated into osteoblast with factors in the transplant site [31]. In any case, it is necessary to transplant MSCs with the cell scaffolds.

## 5. Cell Scaffolds for Bone Tissue Regeneration Using MSCs

Ceramics, synthetic polymers, biopolymers, and their composites have been developed as bone regeneration scaffolds and have been used as framework for cultivation of MSCs. Representative scaffolds are listed in Table 2.

### 5.1. Ceramics-Based Scaffolds

Calcium phosphate, which is the major inorganic component of bone, is widely used as a scaffold for bone differentiation from MSC. Promoted differentiation of MSC to osteoblast by calcium ions and osteoconductivity themselves are expected. For example, when ASCs were transplanted with granular β-TCP into bone defects after osteogenic differentiation, and transplanted to a bone defect site, successful integration of the cell-scaffold construct to the host skeleton was observed [32]. Porous HAp were used as scaffold for bone regeneration using bone marrow derived MSCs [33]. Induction of osteogenic differentiation using dexamethasone increased transplantation efficiency. Biphasic calcium phosphates (BCP) composed of HAp and β-TCP were also promising scaffolds for bone regeneration because of its controlled bioavailability and balance between resorption/solubilization [34].

### 5.2. Synthetic Polymers-Based Scaffolds

Synthetic polymers are also widely known as cell scaffolds for bone regeneration. One of the characteristics of synthetic polymers is that their degradability and mechanical properties can be controlled. Poly(lactide-*co*-glycolide) (PLGA) is biodegradable polymers and its scaffolds with high porosity supported for mesenchymal stem cells to differentiate into osteogenic tissue [37]. Poly(caprolactone) (PCL) is a polymeric material that degrades more slowly than PLGA and its scaffolds can support cells for long periods of time [38]. PEG-based amphiphilic block copolymers are bone regeneration scaffolds with precisely controlled structure [39]. Various kinds of amphiphilic domains, such as poly l-lactic acid (PLLA) and PCA were combined with PEG. It was found that the conjugation of PEG results in dramatic changes of the physical and biological properties, such as swelling, hydrolysis, mechanical strength, and protein and cell adhesion of the scaffolds [39].

### 5.3. Collagen and Its Derivatives-Based Scaffolds

Collagen, which is a component of bone is one of strong candidates of scaffold for bone regeneration [40,41]. Using collagen as scaffold, MSCs distribute throughout the scaffold and differentiate, but its poor mechanical stability may be a shortcoming for a future application in bone tissue engineering [40]. In order to solve this problem, gelatin methacryloyl (GelMA) has been developed. GelMA is a monomer to readily photo-crosslink; three-dimensional (3D) gel as scaffold can be obtained by UV light in the presence of photoinitiator [42]. The crosslinked GelMA with controllable viscoelastic and permeation properties could meet the technological requirements for scaffolds.

### 5.4. Inorganic/Organic Composites Scaffolds

Considering the composition and structure of natural bone (Figure 1), inorganic/organic composites have great attention in the field of bone tissue engineering. Ceramics, such as HAp and β-TCP were combined with synthetic and natural polymers. Composite scaffolds were fabricated by solvent-casting [49] electrospinning [47] and in situ gelation [31]. Incorporation of ceramics improve mechanical properties and promote osteogenic differentiation of MSCs [48,49].

### 5.5. Electrospun Silica Nonwoven Fabrics

MSC differentiation into osteogenic tissue has been extensively investigated using scaffolds, such as Hap [51], nonwoven nano, and microfibrous scaffolds of polymers [52,53,54,55,56], and their composites. Factors affecting the osteogenic differentiation of MSCs are classified into two categories. The first one is the interaction between cell and extracellular matrix(ECM). Because 3D nanofibrous structures of Silica nonwoven fabrics (SNFs) have morphological similarities to collagen fibrils, the SNFs enable to promote favorable biological responses for osteogenic differentiation. Furthermore, cultured cells in SNF easily interact with the surrounding cells due to its high interconnectivity. Another factor affecting the osteogenic differentiation of MSCs is the elasticity of substrates. The differentiation of MSCs was strongly affected by the elastic properties of substrates and rigid matrices mimicking collagenous bone prove osteogenic differentiation. Silica is a rigid and elastic substrate; therefore, it may also contribute to promoting the osteogenic differentiation of MSCs.

**Figure 2:**
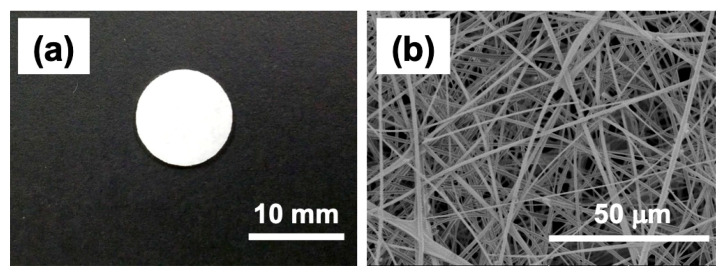
Macroscopic (**a**) and scanning electron microscopic (**b**) images of silica nonwoven fabrics.

The authors’ group has examined bone regeneration from MSC using three-dimensional (3D) silica nonwoven fabric (SNF, Figure 2) [36]. The 3D SNFs were prepared by electrospinning through the sol−gel process [57,58]. Cultured cells embedded in SNFs can migrate and grow in fiber matrix with increasing culture period due to interconnected pores provided by the random fiber orientation. Furthermore, higher mechanical strength prevents shrinkage to help proper cell proliferation, compared with usual polymer nanofiber. Note that interconnected pores provide sufficient permeability for oxygen and nutrients, leading to maintenance of cell viability and function. SNFs have tried to construct coculture systems of fibroblast and hepatocyte [59,60]. To understand contributions of soluble factors to functional enhancement in a hepatocyte-fibroblast coculture, we have constructed the physically separated coculture system using a trans-well culture: rat primary hepatocytes were cultured on the bottom compartment of the trans-well system. SNF cultured with NIH3T3 fibroblasts was placed in the top insert. As a result, the amount of soluble factors secreted from fibroblast cultured in SNF was drastically increased and resulted in the improvement of the functions of cocultured hepatocytes.

**Figure 3:**
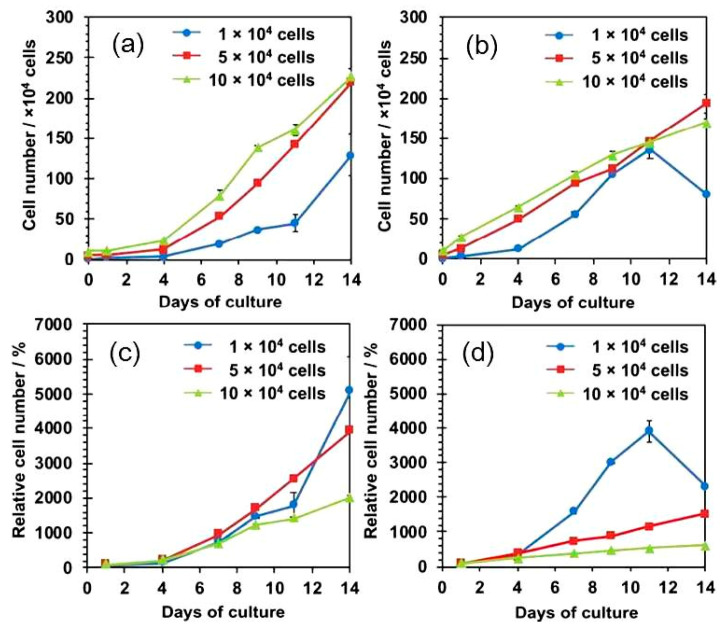
Proliferation profile of human MSCs cultured on three-dimensional (3D) SNFs and tissue culture-treated polystyrene (TCPS) plates with increasing day of culture. (**a**,**b**) cell number on the SNF (**a**) and TCPS plates (**b**) at different seeding densities, 1 (●), 5 (■), and 10 (▲) × 10^4^ cells/well; (**c**,**d**) the ratio of cell number to those after 24 h of culture on SNF (**c**) and TCPS plates (**d**) at different seeding densities, 1 (●), 5 (■), and 10 (▲) × 10^4^ cells/well. (S. E., *n* = 2). ACS Omega, Iijima et al. [36].

**Figure 4:**
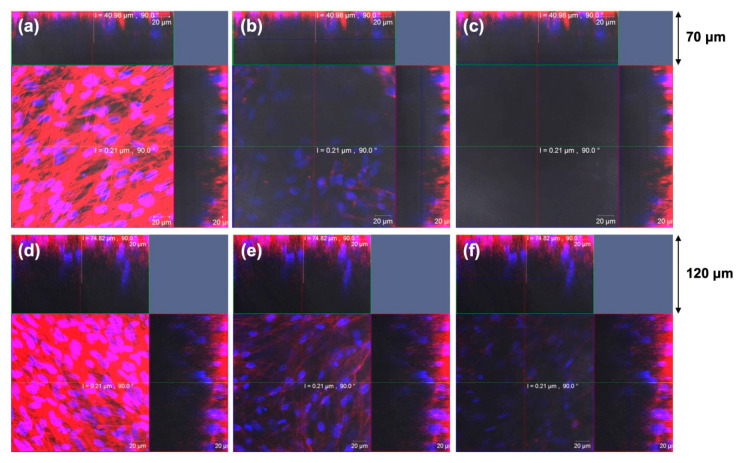
MSC proliferation on SNF was characterized by confocal laser scanning microscopy (CLSM) images after 7 days (**a**–**c**) and 14 days (**d**–**f**) culture at a cell density of 1.5 × 10^5^. Single optical slices are shown near to the SNF surface [1.76 μm, (**a**,**d**)], middle [36.96 μm, (**b**,**e**)], and bottom [66.87 μm, (**c**,**f**)]. Hoechst 33,342 (blue) and Alexa Fluor 594 phalloidin (red) were used to stain cellular nuclei and skeletons, respectively. Scale bars: 20 μm. ACS Omega, Iijima et al. [36].

**Figure 5:**
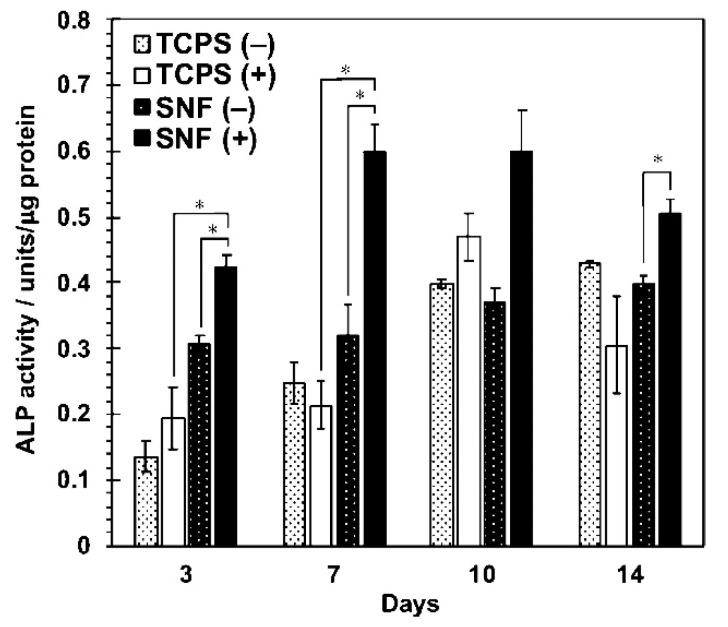
Alkaline phosphatase (ALP) activity of MSCs cultured on SNFs and TCPS plates under the normal medium (−) and the osteogenic differentiation medium (+). Data are shown as the mean ± S. E. (*n* = 4). * indicates significance (*p* < 0.05, Student’s *t*-test). ACS Omega, Iijima et al. [36].

**Figure 6:**
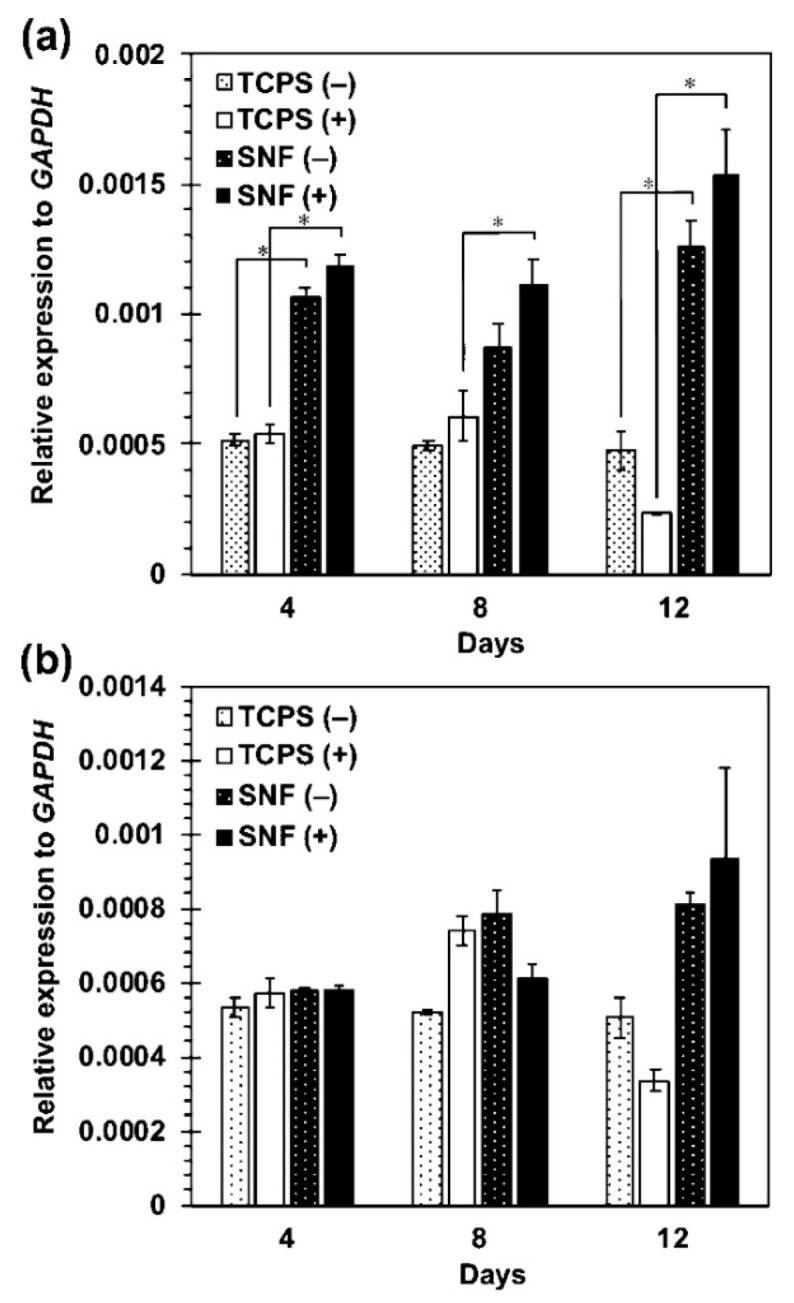
Expression of RUNX2 (**a**) and osteocalcin OCN (**b**) in MSCs cultured on SNFs and TCPS plates under the normal medium (−) and the osteogenic differentiation medium (+). The signal intensity was normalized using that of a control housekeeping gene (human GAPDH gene). Data are shown as the mean ± S. E. (*n* = 2). * indicates significance (*p* < 0.05, Student’s *t*-test). ACS Omega, Iijima et al. [36].

As a representative result, MSCs cultured on the 3D SNF were characterized by the adhesion and proliferation using a WST-8 (2-(2-methoxy-4-nitrophenyl)-3-(4-nitrophenyl)-5-(2,4-disulfophenyl)-2H-tetrazolium) assay, compared with those cultured on conventional 2D tissue culture-treated polystyrene (TCPS) plates. In Figure 3a,b, MSCs cultured on the SNF strikingly grew, in comparison with those on the TCPS plates. The proliferation rate of MSCs describes exactly the difference in cell growth, estimated from the ratio of cell number to those after 24 h of culture on SNF and TCPS plates (Figure 3c,d). At a seeding density of 1 × 10^4^ cells/well, MSCs on the TCPS plates decreased in cell number from day 11 to day 14 due to higher proliferation rate (Figure 3b,d). In contrast, MSCs on the SNF were properly proliferate even after 14 days of culture (Figure 3a,c). Considering these results, the 3D SNF is highly useful for cell scaffold in tissue engineering applications. Confocal laser scanning microscopy (CLSM) was used to show the morphologies of MSCs on SNF. As can be seen in Figure 4, after 7 days of culture, MSCs proliferated at a depth of middle phase (36.96 μm), but not at bottom phase (66.87 μm) (Figure 4b,c). After 14 days of culture, MSCs more proliferated at a middle phase (36.96 μm) than those at 7 days (Figure 4e), and the cells at bottom phase were even more important than those at middle phase (Figure 4f). From these results, MSCs seem to migrate vertically and proliferate in the SNF. This is due to its interconnected pores provided by the random fiber orientation. These features contributed to efficient growth of collected MSCs in vitro are well suitable for bone regeneration, because a large number of MSCs are needed for treatment of large bone defects. When MSCs were cultured in osteogenic differentiation medium, MSCs on SNF showed superior osteogenic differentiation than those on TCPS. As shown in Figure 5, the osteogenic potential of MSCs on SNF was estimated by the alkaline phosphatase (ALP) activity, after culture in an osteogenic differentiation medium for 14 days. MSCs on SNF maintained higher activity of ALP than on the TCPS plates throughout the culture period. This is further suggested by the osteogenic differentiation marker gene, RUNX2, by using quantitative polymerase chain reaction (PCR). The higher expression of RUNX2 on SNF than on the TCPS plate was clearly pronounced in differentiation medium (Figure 6a). This trend is also suggested by the expression of osteocalcin (OCN) (Figure 6b). Moreover, MSC on SNF dominantly differentiated into chondrogenic cell, as compared with conventional spheroid and atelocollagen gel culture [61]. These results suggested that 3D SNFs are potential scaffolds for tissue engineered osteochondral construct, originated from highly porous and elastic SNF characters.

## 6. Future Prospects

Bone regeneration using MSCs is expected to become popular instead of high-risk autologous bone transplantation. To achieve this, it is essential to develop excellent cell scaffolds that proliferate mesenchymal stem cells and efficiently differentiate them into bone. The effects of cell sources and patient age on the bone regeneration using MSCs have not been well analyzed. Development of cell scaffolds optimized for MSCs with different cell sources and conditions is also expected.

## Figures and Tables

**Table 1:** Cell source of mesenchymal cells (MSCs) used for bone tissue regeneration [27].

Cell Source	Advantage	Disadvantage
Bone marrow-derived mesenchymal stem cells (BM-MSCs)	(i) High osteogenic potential	(i) Low abundance
	(ii) Studied extensively	(ii) Highly invasive
Adipose-derived stem cells (ASCs)	(i) High abundant	More studies are needed to test their use in bone repair
	(ii) Easy to harvest surgically	
Umbilical cord mesenchymal stem cells (UC-MSCs)	Lowly invasive	(i) More studies are needed to test their use in bone repair
		(ii) Limited time to harvest
Dental pulp stem cells (DPSCs)	Easy to harvest	More studies are needed to test their use in bone repair

**Table 2:** Representative scaffolds for bone tissue regeneration using MSCs.

Type	Materials	References
Ceramics	β-Tricalcium phosphate (β-TCP)	[32]
	Hydroxyapatite (HAp)	[33]
	Biphasic calcium phosphate (BCP)	[34]
	Bioactive glass	[35]
	Silica nonwoven fabrics (SNF)	[36]
Synthetic Polymers	Poly(lactide-*co*-glycolide) (PLGA)	[37]
	Poly(caprolactone) (PCL)	[38]
	PEG-based amphiphilic block copolymers	[39]
Biopolymers	Type I collagen	[40,41]
	Gelatin methacryloyl (GelMA)	[42]
	Silk fibroin	[43]
	Cellulose	[44]
	Chitosan	[45]
	Chondroitin sulfate	[46]
Composites	HAp/PLGA	[47]
	HAp/Type I collagen	[31]
	β-TCP/Gelatin	[48]
	β-TCP/PCL	[49]
	Chitosan–Gelatin–Chondroitin	[50]
